# Association Between COVID-19 Vaccination, Obesity, and Symptom Burden in a Mexican Population: A Retrospective Cross-Sectional Study

**DOI:** 10.7759/cureus.104332

**Published:** 2026-02-26

**Authors:** Juan M Bravo-Benítez, Victor C Bohórquez, David González- Albarrán, María E Rivera- Castro, Eder P Álvarez- Cortes, César F Pastelin, Carolina Morán

**Affiliations:** 1 Clinical Research, Benemérita Universidad Autónoma de Puebla, Puebla, MEX; 2 Life Sciences, Universidad del Valle de Puebla, Puebla, MEX; 3 Clinical Research, Oaxaca Site Management Organization, Oaxaca, MEX; 4 Life Sciences, Benemérita Universidad Autónoma de Puebla, Puebla, MEX

**Keywords:** clinical symptoms, covid-19 infection, metabolic risk, obesity, sars-cov-2 vaccines

## Abstract

Background

This study aimed to evaluate the association between the presence of COVID-19 symptomatology (quantified as total self-reported symptom count and validated by medical personnel) and obesity in vaccinated and unvaccinated individuals during the epidemiological period from July to December 2021.

Methods

A retrospective, cross-sectional epidemiological study was conducted using data from 2,008 adults with laboratory-confirmed SARS-CoV-2 infection identified during COVID-19 detection campaigns in multiple municipalities of Oaxaca, Mexico, between July and December 2021. Data on age, sex, body mass index (BMI) category, vaccination status, vaccine type, and reported symptoms were analyzed. SARS-CoV-2 infection was confirmed by rapid antigen testing followed by reverse transcription polymerase chain reaction (RT-PCR). The primary outcome was the total number of self-reported symptoms at diagnosis. Statistical analysis included descriptive statistics and one-way ANOVA with Tukey post hoc testing (p<0.05).

Results

Individuals with BMI above normal, particularly those who were unvaccinated and aged over 40 years (age was treated as a stratification variable), reported a higher number of COVID-19 symptoms. Vaccinated patients with elevated BMI showed a lower symptom burden compared with unvaccinated individuals. When analyzed by vaccine type, vaccination with Pfizer-BioNTech (BNT162b2) and CanSinoBio (Ad5-nCoV) was associated with a significantly lower number of reported symptoms, whereas no significant differences were observed for AstraZeneca (ChAdOx1 nCoV-19) or Sinovac (CoronaVac). No significant differences were found between sexes.

Conclusions

Obesity was associated with increased COVID-19 symptom burden in this population-based study. COVID-19 vaccination was associated with fewer reported symptoms among individuals with elevated BMI. Specifically, those vaccinated with Pfizer-BioNTech and CanSinoBio vaccines showed significant associations with reduced symptom burden, whereas vaccines from AstraZeneca and Sinovac did not. These findings support the relevance of vaccination strategies in populations with a high prevalence of obesity and highlight the need to consider metabolic factors and vaccine type when evaluating COVID-19 clinical presentation.

## Introduction

Recently, humanity was confronted by a pandemic caused by the severe acute respiratory syndrome coronavirus 2 (SARS-CoV-2). This novel virus was first identified in late November 2019 in Wuhan, China. The World Health Organization (WHO) declared the outbreak a Public Health Emergency of International Concern on January 30, 2020 [1]. The common routes of transmission of SARS-CoV-2 include: 1) Direct exposure through coughing, sneezing and inhalation of droplets within a range of 1.8 m, and 2) Contact with oral, nasal, and ocular mucous membranes [2]. The clinical manifestations of COVID-19 range from mild to severe, with a substantial number of fatalities. The most frequently reported symptoms include fever, cough, myalgia or fatigue, followed by pneumonia and dyspnea, whereas less common symptoms include headaches, diarrhea, and hemoptysis [3].

SARS-CoV-2 evolved rapidly and spread exponentially worldwide, becoming a major cause of morbidity and mortality. The implementation of large-scale testing to identify individuals infected with COVID-19 was fundamental in limiting further transmission of the disease. In parallel, the scientific community has generated extensive evidence that has improved understanding of the virus, including its molecular characteristics, epidemiological and demographic patterns, and clinical features [4].

Previous studies have shown that the presence of a comorbidity is associated with an increased risk of adverse outcomes, with a three- to four-fold higher probability of developing acute respiratory distress syndrome in patients infected with H7N9 influenza virus [5]. As observed with influenza [6-9], SARS-CoV [10], and the Middle East Respiratory Syndrome Virus (MERS-CoV) [11-14], comorbidities in COVID-19 predispose patients to respiratory failure and death [15-17]. Furthermore, viral infections caused by certain members of Adenoviridae, Herpesviridae, and hepatitis virus families have been reported to be more frequent among individuals with obesity, who in turn, exhibit a higher risk of adverse outcomes during viral epidemics such as influenza and dengue fever [18,19]. A meta-analysis examining the association between obesity and influenza during 2009 pandemic found that, compared with individuals with a normal BMI, the risk of pneumonia increased by 1.33-fold (95% CI:1.05-1.63) in individuals with obesity (BMI ≥30 kg/m²) and by 4.6-fold (95% CI: 2.2-9.8) in those with morbid obesity (BMI >40 kg/m²). In other clinical studies involving patients hospitalized with influenza, obesity was associated with an increased risk of hospital admission [20]. Moreover, morbid obesity (BMI >40 kg/m²) has been linked to approximately a two-fold higher risk of influenza-related mortality (OR = 2.01, 95% CI: 1.29-3.14) [21].

Epidemiological evidence indicates that the prevalence of overweight and obesity has increased in both developed and developing countries. Obesity represents a major public health challenge in Mexico, as it adversely affects the physical, social, and emotional wellbeing of the population. Currently, 75.2% of the Mexican population aged over 20 years is classified as either overweight (39.1%) or obese (36.1%). Consequently, the burden of COVID-19 in Mexico has been substantial, with a higher impact on younger individuals compared with populations in which obesity is less prevalent among young adults [22]. In the context of pandemic mitigation, although Mexico demonstrated a high national COVID-19 vaccine acceptance rate (88%) as reported in a global narrative review [23], the country's persistently elevated obesity burden may still modulate clinical presentation and symptom expression, highlighting the importance of examining COVID-19 outcomes across vaccination and BMI strata.

Among older adults, chronic obstructive pulmonary disease (COPD), cardiovascular diseases (CVD), diabetes mellitus (DM), obesity, and chronic kidney disease (CKD) have been identified as the principal risk factors associated with severe COVID-19 outcomes in Mexican patients [24-27]. In individuals with symptomatic COVID-19 in Mexico, obesity has been reported as the comorbidity most strongly associated with a positive SARS-CoV-2 test result [28].

Nevertheless, the role of obesity as an independent risk factor for COVID-19 severity remains controversial, with debate surrounding outcomes such as hospitalization rates, intensive care unit admission, requirement for mechanical ventilation, and COVID-19-related mortality. One systematic review reported that obesity was not a strong predictor of disease severity nor associated with increased mortality [29], while another found that obesity was associated with greater mortality only in studies with chronic or critical patients [30]. In addition to viral and host-related factors, environmental and social determinants may contribute to these discrepancies [31]. However, these same factors may also complicate the identification of predictors of poor prognosis, as COVID-19 mortality rates appear to correlate with the prevalence of obesity across populations [32-34].

Other studies have pointed out that obese individuals show a deterioration of the immune response to the influenza A virus (IAV) vaccine, as well as to antimicrobial treatments [35,36]. Likewise, it is known that, for H1N1 IAV, obesity can reduce vaccine efficacy [37]. Because of this, the efficacy of other vaccines could also be reduced in obese populations. Although data on COVID-19 vaccine efficacy in obese individuals are not yet available, several studies [35,38,39] have identified relationships between obesity and an altered immune response to vaccination, raising concerns that SARS-CoV-2 vaccines may not be as effective in obese people. These relationships have not yet been conclusively demonstrated [40].

Previous studies examining the relationship between obesity and COVID-19 outcomes have been limited by relatively small sample sizes and single-center designs [41,42]. To address these limitations, we conducted a multicenter study including 2,008 patients from several municipalities in Oaxaca, Mexico. The primary objective was to compare COVID-19 symptom burden between vaccinated and unvaccinated individuals across different BMI categories (normal weight, overweight, and obesity grades I-III). Secondary objectives included: (1) to evaluate whether age and sex modify the association between obesity and symptom burden; and (2) to assess whether the association between vaccination and reduced symptom burden differs by vaccine type.

## Materials and methods

Study design and type

The present study is an observational, retrospective, cross-sectional, and epidemiological study. Vaccination events and the clinical responses of patients were studied without direct intervention from the researchers. The analysis was carried out using data gathered during collective COVID-19 detection campaigns conducted between July and December 2021 in the state of Oaxaca, Mexico. The epidemiological approach allowed the evaluation of the relationship between obesity, vaccination status, and symptomatology associated with infection by SARS-CoV-2. The study was conducted in accordance with the ethical principles outlined in the Declaration of Helsinki and within the epidemiological surveillance framework. Information was collected with verbal consent and handled anonymously, guaranteeing participant confidentiality. The Research Committee, Ethics Committee, and Biosafety Committee of Oaxaca Site Management Organization S.C. (RedOSMO), in accordance with the Mexican General Health Law, NOM-012-SSA3-2012, NOM-220-SSA1-2016, and the International Council for Harmonisation Good Clinical Practice (ICH-GCP) guidelines issued approval for the study (approval no.: CEI-OSMO: 537-A/2021).

Sample population

The sample population was constructed from all patients in whom rapid nasal swab SARS-CoV-2 detection tests were performed in the state of Oaxaca as part of the epidemiological surveillance activities carried out by RedOSMO. The eligible population comprised a total of 2,008 patients, both men and women, aged 18 to 90 years, either vaccinated or unvaccinated against COVID-19, across all BMI categories (normal weight, overweight, and obesity grade 1 (OG1), grade 2 (OG2), and grade 3(OG3), and with or without other comorbidities. Participants were recruited from different municipalities within the state of Oaxaca. Selection was carried out randomly using a randomization algorithm in Microsoft Excel (Microsoft Corp., Redmond, WA, USA). Personal and clinical data were obtained through a direct questionnaire administered by RedOSMO brigade personnel. For the specific assessment of COVID-19-associated symptomatology, a structured symptom checklist based on the epidemiological surveillance criteria established by the World Health Organization was used [43]. This checklist is part of a public health guideline and is freely accessible for use in research. Clinical information was collected using the standardized medical record format established by the Mexican Ministry of Health, in accordance with Official Mexican Standard NOM-004-SSA3-2012 [44]. This official standard is a public regulatory document with unrestricted use for clinical and research purposes within the Mexican healthcare system. Health-related quality of life was assessed using the validated Mexican version of the SF-36 Health Survey [45], a generic instrument freely available for research use, under the standard terms of the MOS Trust (Medical Outcomes Trust), which has demonstrated reliability and validity in the Mexican population

Study groups and stratification

All analyses were performed on patients with a confirmed COVID-19 diagnosis. For analytical purposes, the study population was stratified into several comparison groups according to BMI [46], vaccination status, sex, age, and vaccine type. First, patients were classified according to vaccination status as either vaccinated (W/V) or unvaccinated (WO/V). Within each vaccination group, participants were further stratified by BMI category into normal BMI, underweight, overweight, and OG1, OG2, and OG3, allowing the comparison of COVID-19 symptom counts across different degrees of adiposity. Additionally, BMI categories were analyzed according to sex (male and female) and vaccination status to evaluate potential sex-related differences in symptomatology.

Age-based analyses were performed by stratifying patients into predefined age groups (18-30, 31-40, 41-50, 51-60, and >60 years), which were then compared according to BMI category and vaccination status to assess the interaction between age, obesity, and COVID-19 symptom burden. Furthermore, the number of reported symptoms was compared across different vaccine types, including AstraZeneca, Sinovac, Pfizer, and CansinoBio, with WO/V patients serving as the reference group.

Finally, subgroup analyses were conducted for each vaccine brand to evaluate symptomatology across BMI categories among W/V and WO/V patients. These analyses allowed the assessment of vaccine-specific effects on COVID-19 symptoms in individuals with normal BMI, overweight, and varying degrees of obesity. This multilevel stratification enabled a comprehensive evaluation of the relationship between obesity, vaccination status, demographic factors, and COVID-19 symptom expression.

Standardized nomenclature

Throughout the manuscript and figures, standardized nomenclature was applied to ensure clarity and consistency. Body mass index is referred to as BMI and categorized as normal BMI, underweight, overweight, and obesity grades 1-3. Vaccination status is consistently indicated as W/V or WO/V, while sex is denoted as males (M) and females (F). The outcome variable, number of COVID-19 symptoms, represents the total count of self-reported symptoms at the time of diagnosis. Vaccine types are identified by their commercial names.

Anthropometric evaluation

Anthropometric evaluation was carried out by calculating BMI, which is obtained by dividing the subject’s body weight in kilograms by the square of their height in meters. According to World Health Organization criteria, overweight was defined as a BMI greater than or equal to 25 kg/m², and obesity as a BMI greater than or equal to 30 kg/m². Weight and height measurements were obtained using a mechanical column scale (BAME, Model 420, Mexico) with a capacity of 140 kg and a precision of 0.1 kg, as well as a stadiometer with a precision of 0.1 cm. Both instruments were calibrated between measurements.

SARS-CoV-2 detection

SARS-CoV-2 infection was initially detected using a rapid antigen test based on nasal swab sampling. Specimens were collected using medium nasal swabs and processed with the SARS-CoV-2 Rapid Antigen Test (Roche Diagnostics GmbH, Mannheim, Germany), strictly following the manufacturer’s instructions. All positive rapid antigen test results were subsequently confirmed by reverse transcription polymerase chain reaction (RT-PCR), which was used as the confirmatory diagnostic method. Molecular confirmation was performed using the ExProbe™ SARS-CoV-2 Testing Kit (Catalogue number: 68020, TBG Biotechnology Corp., USA), a real-time RT-PCR assay authorized for emergency use and widely implemented in clinical laboratories in Mexico during the COVID-19 pandemic.

RT-PCR assays were carried out according to the manufacturer’s protocol. The ExProbe™ kit targets conserved regions of the RdRP, N, and E genes of SARS-CoV-2, allowing qualitative detection of viral RNA. Amplification and fluorescence detection were performed using a real-time PCR system compatible with the assay specifications. Appropriate positive and negative controls supplied with the kit were included in each run to ensure assay validity. Only patients with confirmed positive RT-PCR results were included in the final analysis.

Vaccination status

Vaccination status was determined through direct questioning and, when available, verification of official vaccination records. Patients were classified as W/V or WO/V. W/V patients were further categorized according to the type of vaccine received. The vaccines included in the analysis were: BNT162b2 (Pfizer-BioNTech, Pfizer Inc., New York, USA / BioNTech SE, Mainz, Germany); Ad5-nCoV (CanSino Biologics Inc., Tianjin, China); ChAdOx1 nCoV-19 (AstraZeneca/Oxford, AstraZeneca PLC, Cambridge, United Kingdom); and CoronaVac (Sinovac Biotech Ltd., Beijing, China). Vaccines with a low frequency of administration in the study population, such as Moderna (mRNA-1273) and Sputnik V (Gam-COVID-Vac), were not included in the statistical analysis due to insufficient sample size.

Symptomatology evaluation

COVID-19-associated symptomatology was evaluated using a structured questionnaire applied at the time of diagnosis by trained medical personnel. For each patient, the presence or absence of the following symptoms was recorded: fever, cough, myalgia (muscle pain), fatigue, pneumonia (confirmed by clinical or radiological findings), dyspnea (shortness of breath), headache, diarrhea and hemoptysis (coughing up blood). To ensure data reliability, all reported symptoms were clinically corroborated by the attending medical staff during the evaluation. This process also served as a quality filter: from an initial pool of approximately 20,000 individuals screened during the COVID-19 detection campaigns, only 2,008 patients with complete and verified clinical data were included in the final analysis. The total number of symptoms reported by each patient was tallied and used as the primary outcome variable.

Statistical analysis

Statistical analysis was carried out using GraphPad Prism version 8.1 (GraphPad Software, San Diego, CA, USA). This software was used under an institutional license held by Benemérita Universidad Autónoma de Puebla, Mexico. For variables such as age, body mass index, gender, and number of symptoms, descriptive statistics were performed (mean and standard error of the mean). Qualitative variables such as gender, vaccination status, and type of vaccine administered were analyzed using absolute frequencies and percentages. Before the application of inferential tests, the normality of the distribution of quantitative variables was evaluated using the Shapiro-Wilk test, with the objective of verifying the suitability of parametric tests. Since the variables of interest showed distributions compatible with normality and considering the sample size, parametric tests were used. For comparisons of the number of symptoms between two or more independent groups defined by vaccination status, BMI category, age group, gender, and type of vaccine received, a one-way analysis of variance (ANOVA) was performed. This test allowed the identification of overall differences in the number of symptoms among the analyzed groups. In cases in which the ANOVA showed statistically significant differences, a Tukey post hoc multiple-comparison test was applied to identify the specific groups between which significant differences were present. Additionally, for the analysis of relationships between categorical variables, such as gender and vaccination status, chi-square (χ²) tests were performed when deemed relevant. All results were expressed as means ± standard error of the mean, and a significance level of p <0.05 was established for all tests.

## Results

For the present study, a total of 2,008 patients, with or without previous vaccination but with established obesity as a comorbidity, were analyzed. Sampling brigades were distributed as follows: Oaxaca de Juárez (1,678 patients), Miahuatlán de Porfirio Díaz (205 patients), Candiani (12 patients), San Bartolo Coyotepec (72 patients), and Zimatlán de Álvarez (41 patients). For analytical purposes, the population was divided as follows: 59% W/V (1,175 patients) and 41% WO/V (830 patients). Of the total population, 44% were female patients (893 participants), and 56% were male patients (1,115 participants), indicating a trend of COVID-19 disease in the male population. With respect to vaccination status stratified by gender, 26% (513 patients) were W/V men, 33% (662 patients) were W/V women, 19% (450 patients) were WO/V men, and 22% (450 patients) were WO/V women. To analyze the number of symptoms by age range, confidence intervals were used, as the largest proportion of patients was in the 18-30-year age group (38%), followed by the 31-40-year group (28%), 41-50 years (17%), 51-60 years (9%), and >60 years (8%), respectively.

For both male and female participants, the age group with the highest number of patients was 18-30 years (30% (340 patients) and 37% (414 patients), respectively). The mean BMI for the study population was 27.8 kg/m², including both men and women. The majority of men were classified as overweight (45%; 404 patients), as were the majority of women (41%; 454 patients). Vaccine brands were distributed as follows: AstraZeneca was administered to 40% of W/V patients, followed by CansinoBio and Pfizer, each accounting for 22%. Sinovac was reported in 16% of patients, while Moderna and Sputnik V were not included in the statistical analysis due to their low usage in the state of Oaxaca. With respect to gender, most male patients were vaccinated with AstraZeneca (42%), followed by Pfizer (21%), CansinoBio (18%), Sinovac (18%), and Sputnik V (1%). Among female patients, the highest proportions were AstraZeneca (39%), followed by CansinoBio (24%), Pfizer (23%), and Sinovac (14%).

**Figure 1:**
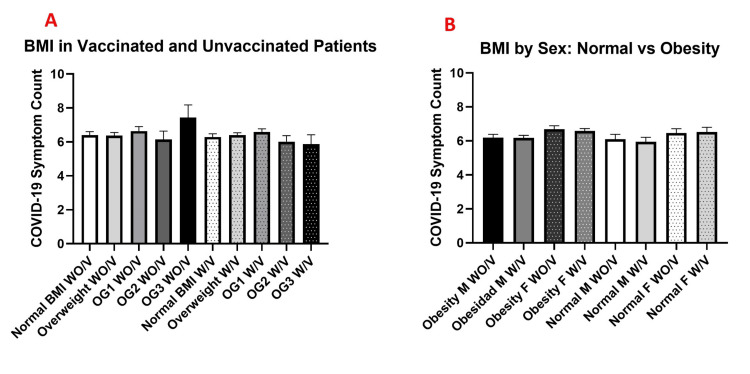
Number of COVID-19–related symptoms according to body mass index (BMI) category and vaccination status Mean number of COVID-19–related symptoms according to body mass index (BMI) category and vaccination status (A) and stratified by sex and BMI status (B). Panel A shows comparisons between unvaccinated (WO/V) and vaccinated (W/V) individuals across BMI categories: normal weight, overweight, and obesity grade 1 (OG1), grade 2 (OG2), and grade 3 (OG3). Panel B presents symptom counts in men (M) and women (F) with normal BMI or obesity, further subdivided by vaccination status. Data are presented as mean ± standard error of the mean (SEM). Statistical analysis was performed using one-way ANOVA followed by Tukey’s post hoc test, and no statistically significant differences were detected between groups. (COVID-19 symptoms were assessed using the standardized checklist described in [43]; BMI classification was based on WHO criteria [46]).

For the analysis of the number of symptoms reported by W/V compared with WO/V across different degrees of obesity (BMI), statistical analysis showed no significant differences in the number of symptoms with respect to patients’ BMI (p >0.05), although the highest number of symptoms was observed in WO/V patients with grade III obesity (7.4 ± 0.75 SEM) (Figure 1A).

When comparing men and women with obesity and normal BMI who were either W/V or WO/V, the analysis showed no significant differences between genders in terms of the number of symptoms reported by patients with BMI above normal (p>0.05) (Figure 1B).

**Figure 2:**
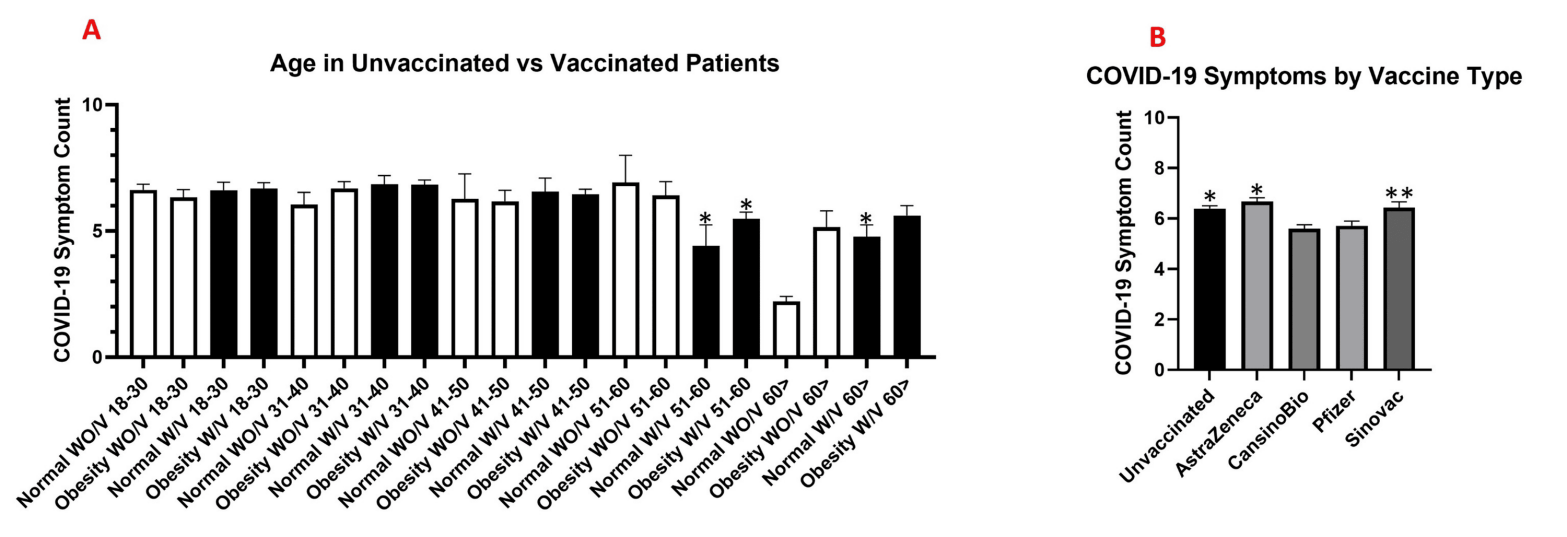
Number of COVID-19 symptoms reported by vaccinated and unvaccinated individuals Number of COVID-19 symptoms reported by vaccinated (W/V) and unvaccinated (WO/V) individuals across age groups (A) and by vaccine type (B). (A) Mean COVID-19 symptom counts were compared between vaccinated and unvaccinated participants stratified by age groups (18–30, 31–40, 41–50, 51–60, and >60 years) and body mass index (BMI) category (normal weight and obesity). Asterisks indicate statistically significant differences compared with Obesity WO/V 31-40 group (*p<0.05). (B) Comparison of COVID-19 symptom burden among unvaccinated individuals and those vaccinated with AstraZeneca (ChAdOx1 nCoV-19), Sinovac (CoronaVac), Pfizer-BioNTech (BNT162b2), and CanSinoBio (Ad5-nCoV). *p<0.05 vs. CansinoBio and vs. Pfizer; **p<0.05 vs. CansinoBio. Data are presented as mean ± standard error of the mean (SEM). Statistical analysis was performed using one-way analysis of variance (ANOVA) followed by Tukey’s multiple comparisons test.

Regarding the number of symptoms across different age intervals, a significant difference (p<0.05) was observed between WO/V obese patients in the 31-40 year age group (6.834 ± 0.189 SEM) and patients aged over 60 years with a normal BMI who had received any type of vaccine (4.77 ± 0.46 SEM). Similarly, significant differences in the number of reported symptoms were found between WO/V obese patients in the 31-40-year age group (6.834 ± 0.187 SEM) and W/V patients in the 51-60-year age group (5.478 ± 0.65 SEM) (p<0.05) (Figure 2A).

With respect to vaccine brand and the reduction in the number of symptoms among obese patients, a significant difference was observed in the number of symptoms reported by COVID-19-positive patients compared with those who did not receive any vaccine (WO/V) (p<0.0001). Patients vaccinated with CansinoBio (5.592 ± 0.160 SEM) and Pfizer (5.71 ± 0.1846 SEM) reported fewer symptoms than the WO/V group (6.394 ± 0.1142 SEM) (p<0.05), whereas patients vaccinated with Sinovac (6.434 ± 0.2296 SEM) and AstraZeneca (6.67 ± 0.149 SEM) showed no significant differences (p>0.05) (Figure 2B).

**Figure 3:**
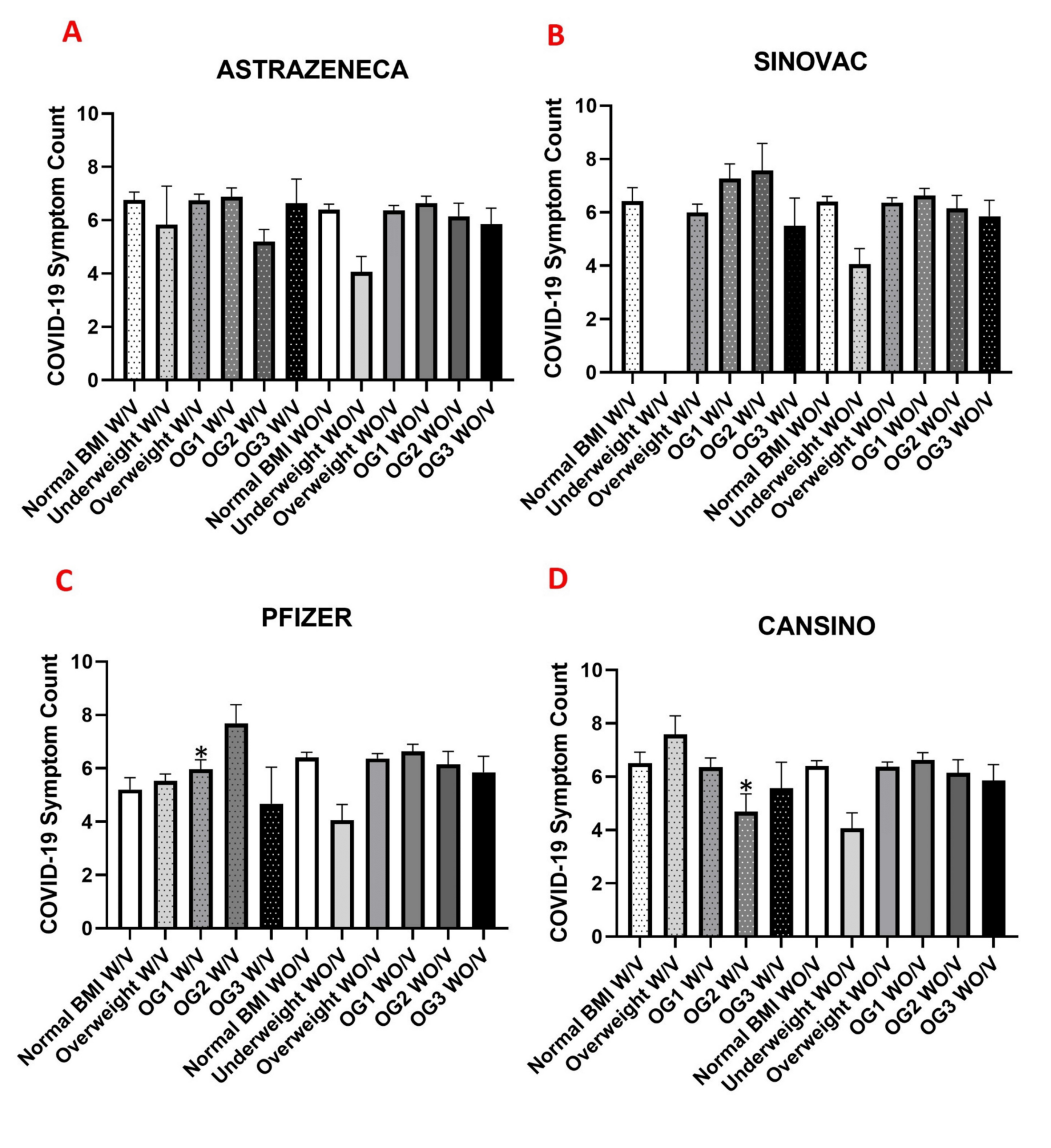
COVID-19 symptoms, stratified by the vaccine brand Number of COVID-19 symptoms reported during the study period (July–December 2021), stratified by the vaccine brand: (A) AstraZeneca (ChAdOx1 nCoV-19), (B) Sinovac (CoronaVac), (C) Pfizer–BioNTech (BNT162b2), and (D) CansinoBio (Ad5-nCoV). Within each panel, symptom counts are compared between vaccinated (W/V) and unvaccinated (WO/V) individuals across body mass index (BMI) categories: normal weight, overweight, and obesity grades I (G1), II (G2), and III (G3). Data are expressed as mean ± standard error of the mean (SEM). Statistical significance was assessed using one-way ANOVA followed by Tukey’s post hoc test. Asterisks indicating significant differences between vaccinated and unvaccinated individuals within the same BMI category (*p<0.05). (COVID-19 symptoms were assessed using the standardized checklist described in [43]; BMI classification was based on WHO criteria [46]).

When analyzing patient data stratified by vaccine brand, patients vaccinated with AstraZeneca (Figure 3A) and Sinovac (Figure 3B) across different degrees of obesity showed no significant differences in the number of COVID-19 symptoms (p>0.05).

In patients vaccinated with the Pfizer brand, a significant difference in the number of COVID-19 symptoms was observed between overweight WO/V patients and W/V patients with different degrees of obesity, indicating a reduction in symptom number among W/V individuals (5.2 ± 0.25 SEM vs 7.7 ± 0.7 SEM, p<0.05) (Figure 3C). Similarly, among patients vaccinated with the CanSino brand, a significant difference was found between W/V obese patients and WO/V patients with different degrees of obesity (4.7 ± 0.6 vs 6.6 ± 0.20 SEM, p<0.05) (Figure 3D).

## Discussion

The results obtained in this study associate high BMI in obese COVID-19 patients with complications related to pneumonia, as well as with a greater number of COVID-19 symptoms in WO/V patients with BMI above normal, aged over 41 years. These findings corroborate that older age and higher BMI are positively associated with the risk of COVID-19 complications, in line with what has been reported by other authors [20].

The role of obesity as a risk factor in COVID-19 remains controversial [31]. Evidence regarding obesity as a risk factor for severe COVID-19 outcomes has been nuanced. Zhou et al. [29] reported that obesity is a high-risk factor for severe disease, while Mesas et al. [30] found that obesity was associated with increased mortality, although this association was significant only in studies with fewer chronic or critical patients. While Hernández-Garduño [28] reported that obesity is the comorbidity most strongly associated with a positive SARS-CoV-2 test result in Mexico, our study extends this observation by demonstrating that among infected individuals, obesity is also associated with a higher symptom burden. Moreover, in Mexican populations, advanced age and comorbidities such as COPD, CVD, DM, obesity, and CKD have been consistently identified as major risk factors for severe COVID-19 [28,32-34]. Beyond host biological factors, environmental and social determinants, including air pollution, population density, ethnicity, discrimination, and structural inequalities within healthcare systems, may contribute to variability in COVID-19 outcomes across populations [30]. However, these same contextual factors can also complicate the identification of independent predictors of disease severity and mortality [32-34].

Other studies have shown that obese individuals have a deteriorated immune response to the influenza A virus vaccine and to antimicrobial treatments [35,36]. It is also known that, in the case of influenza A virus H1N1, obesity may be associated with reduced vaccine efficacy [37], which suggests that vaccine efficacy against other diseases could also be reduced in obese populations. Although data on COVID-19 vaccine efficacy in obese individuals are not yet available, our study shows that there is a significant difference in COVID-19 symptom reduction among W/V patients with BMI above normal. However, the reduction in symptomatology appears to depend on the type of vaccine administered, with the CanSino and Pfizer vaccines showing greater effectiveness in obese patients [47]. The analysis of the relationship between patient gender and symptomatology indicates that gender is not a determining factor in the reduction of COVID-19 symptoms, as no significant differences were found between patients with BMI above normal of either gender. On the other hand, previous studies have had small sample sizes as one of their main limitations, as well as sampling conducted at a single location [48]. The population in our study was multicenter and distributed across different sites in the state of Oaxaca, where sampling took place. However, the study still needs to be expanded to other states in the country, and the analysis of other SARS-CoV-2 variants must be continued to further investigate the relationship between vaccination, symptomatology, and obesity in the Mexican population. It is also necessary to include additional bench studies to assess not only the presence of the virus in positive rapid test results, but also biochemical modifications and patient immunogenicity, such as interleukin profiles associated with more severe virus-related complications, to improve diagnosis and the management of SARS-CoV-2-infected patients [49].

Given the cross-sectional design of this study, all findings should be interpreted in terms of associations rather than causal relationships. While we observed consistent patterns, such as higher symptom burden in WO/V individuals with elevated BMI and significant associations with specific vaccine types, these results do not imply that obesity directly causes increased symptomatology or that vaccination directly reduces it. Unmeasured confounding variables (e.g., socioeconomic status, access to healthcare, baseline health status) may influence both exposure and outcome. Therefore, we have carefully framed our conclusions within the limits of association-based inference, and we encourage readers to view these findings as hypothesis-generating rather than definitive.

This study has several limitations. First, its cross-sectional design precludes causal inferences; therefore, all findings should be interpreted as associations rather than causal relationships. Second, although symptom data were initially collected through self-report, all symptoms were clinically corroborated by trained medical personnel at the time of diagnosis, reducing recall bias. Additionally, this verification process served as a quality filter: from approximately 20,000 screened individuals, only 2,008 patients with complete and verified data were included. However, objective clinical measures (e.g., oxygen saturation, inflammatory markers) were not available. Third, we did not perform multivariate regression analyses to adjust for potential confounders (e.g., socioeconomic status, comorbidities beyond obesity), which limits the ability to isolate the independent effect of obesity. Furthermore, although metabolic disorders such as diabetes could theoretically confound the association between obesity and symptom burden, only 147 patients (7.3% of the study population) had confirmed diabetes. This relatively low prevalence suggests that the observed association between elevated BMI and increased COVID-19 symptomatology is not predominantly driven by diabetes in this sample. Fourth, the study was conducted solely in Oaxaca, Mexico, which may limit generalizability to other populations. Data on SARS-CoV-2 variants were not available, and vaccination status relied on self-report or record verification, with potential misclassification bias. Despite these limitations, the large sample size, multicenter design, clinically corroborated symptom data, and detailed BMI stratification provide valuable real-world evidence on the relationship between obesity, vaccination, and COVID-19 symptomatology.

## Conclusions

Based on our findings, gender does not appear to be a determining factor in COVID-19 symptomatology, as no significant differences were observed between obese male and female patients. Age, however, is associated with COVID-19 symptom burden: among unvaccinated individuals, young adults aged 18-29 years with elevated BMI reported a higher number of symptoms compared with older age groups. This observation may be partially explained by the higher prevalence of BMI above normal within this younger population, although further research is needed to confirm this hypothesis. Furthermore, our results indicate that vaccination is associated with a reduction in COVID-19 symptom burden among individuals with elevated BMI, although this effect varies according to the type of vaccine administered. Certain vaccine platforms (Pfizer-BioNTech and CanSinoBio) demonstrated greater effectiveness in reducing symptomatology in patients with obesity, highlighting the importance of considering metabolic status when designing and implementing vaccination strategies. Overall, these findings underscore the need for targeted public health approaches aimed at populations with increased metabolic risk.
